# DNA Base Damage Repair Crosstalks with Chromatin Structures to Contract Expanded GAA Repeats in Friedreich’s Ataxia

**DOI:** 10.3390/biom14070809

**Published:** 2024-07-08

**Authors:** Yanhao Lai, Nicole Diaz, Rhyisa Armbrister, Irina Agoulnik, Yuan Liu

**Affiliations:** 1 Department of Chemistry and Biochemistry, Florida International University, 11200 SW 8th Street, Miami, FL 33199, USA; yalai@fiu.edu (Y.L.); nicolediaz00@outlook.com (N.D.); 2 Biochemistry Ph.D. Program, Florida International University, 11200 SW 8th Street, Miami, FL 33199, USA; rarmbris@fiu.edu (R.A.); ira.agoulnik@gmail.com (I.A.); 3 Biomolecular Sciences Institute, Florida International University, 11200 SW 8th Street, Miami, FL 33199, USA

**Keywords:** base excision repair, inhibition of histone H3K9 methylation, trinucleotide, repeat contraction, Friedreich’s ataxia

## Abstract

Trinucleotide repeat (TNR) expansion is the cause of over 40 neurodegenerative diseases, including Huntington’s disease and Friedreich’s ataxia (FRDA). There are no effective treatments for these diseases due to the poor understanding of molecular mechanisms underlying somatic TNR expansion and contraction in neural systems. We and others have found that DNA base excision repair (BER) actively modulates TNR instability, shedding light on the development of effective treatments for the diseases by contracting expanded repeats through DNA repair. In this study, temozolomide (TMZ) was employed as a model DNA base damaging agent to reveal the mechanisms of the BER pathway in modulating GAA repeat instability at the frataxin (*FXN*) gene in FRDA neural cells and transgenic mouse mice. We found that TMZ induced large GAA repeat contraction in FRDA mouse brain tissue, neurons, and FRDA iPSC-differentiated neural cells, increasing frataxin protein levels in FRDA mouse brain and neural cells. Surprisingly, we found that TMZ could also inhibit H3K9 methyltransferases, leading to open chromatin and increasing ssDNA breaks and recruitment of the key BER enzyme, pol β, on the repeats in FRDA neural cells. We further demonstrated that the H3K9 methyltransferase inhibitor BIX01294 also induced the contraction of the expanded repeats and increased frataxin protein in FRDA neural cells by opening the chromatin and increasing the endogenous ssDNA breaks and recruitment of pol β on the repeats. Our study provides new mechanistic insight illustrating that inhibition of H3K9 methylation can crosstalk with BER to induce GAA repeat contraction in FRDA. Our results will open a new avenue for developing novel gene therapy by targeting histone methylation and the BER pathway for repeat expansion diseases.

## 1. Introduction

Trinucleotide repeat (TNR) expansion is the cause of more than 40 neurodegenerative diseases, including Huntington’s disease (HD)(CAG/CTG), Friedreich’s ataxia (FRDA)(GAA/TTC), fragile X syndrome (FXS)(CGG/CCG), and myotonic dystrophy (DM)(CTG/CAG), among others [1,2,3]. A study in 2023 has identified a new type of TNR expansion neurodegenerative disease, late-onset cerebellar ataxia (LOCA), also named GAA-FGF14 ataxia/spinocerebellar ataxia 27B, that is caused by GAA/TTC repeat expansion at intron 1 of the fibroblast growth factor 14 (FGF14) gene [4]. There are no effective treatments for TNR expansion diseases due to the inherited expanded TNR in the patient genome and poor understanding of molecular mechanisms underlying somatic TNR instability in neural systems. With the discovery of more TNR expansion diseases, this group of neurodegenerative diseases can exhibit significant effects in compromising the health and life quality of patients. Among the TNR expansion diseases, FRDA is the one that can result in multiple dysfunctions, including neurodegeneration, diabetes, and cardiomyopathy, which is the cause of death.

Friedreich ataxia (FRDA) is a progressive autosomal genetic neurodegenerative disease that affects one in 50,000 Caucasians [5,6,7,8]. It is the most common hereditary ataxia, characterized by neural degeneration, spasticity, dysarthria, loss of motor reflexes [7,9,10], cardiomyopathy, and diabetes [11]. FRDA patients suffer from a severe decline in muscle coordination, causing a progressive deterioration in life quality [7,10] and a shortened life expectancy [7,9,10,12]. FRDA is caused by *FXN* gene silencing in patients resulting from the expanded GAA repeats in the first intron of the gene [13,14,15,16]. Fxn protein deficiency in the mitochondria can disrupt iron-sulfur cluster assembly and energy production, causing oxidative stress, oxidative DNA damage, reduction of DNA repair capacity, gene mutations, and apoptosis [8,9,17,18,19]. Presently, there are no effective treatments for FRDA [8,10,20,21,22] because of expanded GAA repeats in the patient’s genome, and lack of understanding of the molecular mechanisms underlying somatic instability, i.e., contraction or expansion of the expanded GAA repeats in neural cells and systems.

It has been shown that *FXN* gene silencing can result from heterochromatinization featured with a high level of H3K9 di- and tri-methylation (H3K9me2/3) and a low level of H3K9 acetylation at the *FXN* gene [23,24] caused by the expanded GAA repeats that can also form triplex structures [25,26,27] during *FXN* gene transcription. The epigenetic alterations and secondary structures on the expanded GAA repeats prevent RNA polymerases from synthesizing RNA during *FXN* gene transcription [9,10,18,28,29]. Thus, it is conceivable that shortening of the expanded GAA repeats and alleviation of heterochromatinization can mitigate the neurodegenerative phenotypes of FRDA. This notion is supported by the fact that the inhibition of histone deacetylase activity can increase histone acetylation at the *FXN* gene, resulting in euchromatin and relief of *FXN* gene silencing in FRDA models [30,31]. It is further supported by a recent study showing that inhibitors of H3K9 and H3K27 methyltransferases, BIX01294, and GSK126 in FRDA patient fibroblasts disrupted heterochromatin, increasing Fxn protein at the mRNA level [32]. Moreover, a study by the Wade-Martins group demonstrated that the inhibition of SUV4-20H1 and SUV4-20H2 methyltransferase activity by the compound A-196 and its derivatives can reduce H4K20me2/3 and stimulate *FXN* gene expression at both the mRNA and protein levels in FRDA patient fibroblasts [33], providing evidence that the suppression of H4K20me2/3 may also lead to open chromatin. However, it remains challenging to develop approaches to disrupt GAA repeat triplexes and shorten the expanded GAA repeats due to a poor understanding of the molecular mechanisms underlying somatic expansion and contraction of repeated DNA sequences through the processing of their secondary structures by DNA metabolic enzymes in neural cells and systems.

Previous studies have indicated that TNRs can be contracted by γ-irradiation, DNA replication inhibitors, and several chemical compounds via double-strand DNA breaks, DNA replication fork stalling, DNA crosslinks, and modification of DNA bases in animal cells and human patient lymphoblasts [34,35]. These findings suggest that somatic repeat contraction can be induced by DNA damage through DNA repair. The studies from our group have further demonstrated that the BER pathway plays a pivotal role in mediating TNR contraction and preventing repeat expansion through different mechanisms via the coordination among BER enzymes and cofactors [36,37,38,39,40,41]. Moreover, we have found that alkylated DNA bases on the expanded GAA repeats at the *FXN* gene induced by a model DNA alkylating agent, temozolomide (TMZ), can lead to the contraction of expanded repeats through BER in FRDA patient lymphoblasts [38]. We demonstrate that TMZ-induced alkylated DNA bases in the expanded GAA repeats are removed by the BER pathway through which single-strand DNA (ssDNA) breaks are generated. Subsequently, this results in the formation of TTC loops on the template that are skipped by DNA polymerase β (pol β), thereby leading to the contraction of expanded repeats [38]. We further hypothesize that TMZ can also induce alkylated DNA bases on the expanded GAA repeats at the *FXN* gene in FRDA neural cells and tissue, causing repeat contraction through BER, leading to the disruption of heterochromatin and stimulation of *FXN* gene expression. We tested the hypothesis by examining the effects of TMZ on the GAA repeat contraction, chromatin structures, and *FXN* gene expression in FRDA transgenic mouse neuronal cells and brain tissue and human FRDA neural cells differentiated from FRDA patient pluripotent stem cells (iPSCs). We found that TMZ led to the contraction of expanded GAA repeats at the *FXN* gene in FRDA transgenic mouse brain tissue and differentiated FRDA neural cells. We further demonstrated that TMZ suppressed heterochromatinization on the expanded GAA repeats of the *FXN* gene by inhibiting histone H3 lysine 9 (H3K9) methyltransferase activity. Consequently, this led to the opening of the chromatin on the expanded repeats, the accumulation of single-strand DNA (ssDNA) breaks resulting from damaged DNA bases, the recruitment of pol β on the repeats, and stimulation of *FXN* gene expression in FRDA transgenic mouse tissues. Our results indicated that TMZ simultaneously disrupted heterochromatinization and induced the BER pathway that contracted the expanded GAA repeats, increasing *FXN* gene expression. The results further suggested that the interplay between BER and the opening of chromatin structures can cause large contraction of the expanded GAA repeats at the *FXN* gene in FRDA neural cells. Our studies suggest that an effective therapeutic strategy for FRDA can be developed by contracting the expanded GAA repeats and upregulating *FXN* gene expression through targeting BER and chromatin structures.

## 2. Materials and Methods

### 2.1. Materials

PCR primers and barcode DNA oligonucleotides were synthesized by Integrated DNA Technologies (Coralville, IA, USA). All other chemicals were from ThermoFisher Scientific (Waltham, MA, USA) and Sigma-Aldrich (St. Louis, MO, USA).

### 2.2. Breeding of FRDA Transgenic Mice

The YG22R (*Fxn^tm1Mkn^* Tg (*FXN*) YG22Pook/J) FRDA transgenic mice developed by the Pook group [12,42] were used to determine the effects of TMZ on the GAA repeat stability and *FXN* gene expression. The mice expressed the human *FXN* gene and exhibited the major phenotypes of FRDA. In particular, the YG22R FRDA mice exhibited epigenetic and oxidative stress phenotypes in neurons and cardiomyocytes like FRDA patients [42,43]. Two breeding pairs of transgenic mice homozygous for mouse *Fxn* gene knockout (*Fxn*^−^) and hemizygous of a single copy of human *FXN* YAC transgene containing one sequence of ~190 GAA repeats were purchased from the Jackson Laboratory (Bar Harbor, ME, USA). Mice underwent husbandry in the Florida International University (FIU) Animal Core Facility under protocol # IACUC-17-017-CR02, approved by the FIU IACUC Committee. The FRDA breeding mice were bred to C57BL/6J mice, and subsequent mouse lines were crossed to generate FRDA transgenic mice according to the instructions from the Jackson Laboratory.

### 2.3. FRDA Mouse Genotyping

Mice were genotyped with PCR to identify FRDA homozygotes. Mouse ear tissues were obtained and incubated in 100 μL of 50 mM NaOH for 1 h at 95 °C, allowing the release of genomic DNA. Subsequently, 15 μL of 1 M Tris HCl (pH 8.0) was added to neutralize the DNA samples. The DNA samples were then subjected to centrifugation at 14,000 rpm for 5 min. The supernatant was collected, and DNA concentrations were measured by the NanoDrop 2000 (ThermoFisher Scientific, Waltham, MA, USA). Isolated genomic DNA (400 ng total DNA) was used for PCR amplification using the DreamTaq master mixture kit (ThermoFisher, Waltham, MA, USA) with wild-type forward primer, 5′-CTT CCC TCT ACC CTG CCT TC-3′, mutant forward primer, GCC TGA AGA ACG AGA TCA GC, and common reverse primer, GGA GAA CAG TGG ACA CAG TAA CA. The PCR program for genotyping was as follows: 98 °C 3 min, 1 cycle, 98 °C 20 s, 68 °C 30 s, and 72 °C 1.5 min for 40 cycles.

### 2.4. TMZ Treatment for FRDA Transgenic Mice

Twelve-week-old FRDA mice were subjected to treatment of TMZ at 25 mg/kg body weight or vehicle, 0.1% dimethyl-sulfoxide (DMSO) in saline through oral gavage (three times per week) for three months. Five male FRDA mice were used for the TMZ-treated group or vehicle control group. TMZ was dissolved (40 mg/mL) and then diluted (5 mg/mL) in saline. The mice in the control group were treated with 0.1% DMSO diluted in saline. Mice were then euthanized, and their brain tissue was collected and stored at −80 °C. A schematic diagram for experiments using FRDA transgenic mice is illustrated in Supplementary Figure S1. The mouse experiments were conducted in the FIU Animal Core Facility under protocol # IACUC-17-043-CR02, approved by the FIU IACUC Committee.

### 2.5. Primary Culture of Cerebellar Neuronal Cells of FRDA Transgenic Mice and Neuronal Treatment by TMZ

FRDA cerebellar neuronal cells were isolated and cultured as described previously [44]. FRDA mouse pups (three-day-old) were decapitated. The mouse brain was isolated, and the mouse cerebellum was cut off and then cut into 0.5–1 mm pieces using a sterile razor blade. The cerebellar tissue pieces were transferred to a 35 mm culture dish and triturated using a Pasteur pipet and then incubated with 2 mL 0.25% trypsin EDTA solution (Life Technologies/ThermoFisherScientific, Waltham, MA, USA) for 30 min at 37 °C to generate FRDA mouse cerebellum cell suspension. The cell suspension was transferred to a 15 mL conical tube containing 10 mL of neurobasal medium containing B27 supplement and mixed gently. Cell homogenates were centrifuged at 1000 rpm for 4 min. Cells were washed using HBSS without Ca^2+^ and Mg^2+^ twice and cultured in a dish coated by poly-D lysine (Fisher Scientific, Waltham, MA, USA) (50 μg/mL) in MEM-glutamax medium containing 10% fetal bovine serum (FBS), penicillin, and streptomycin at 37 °C and 5% CO_2_ for 24 h. FRDA mouse neural cells were then washed with HBSS and cultured in neurobasal medium containing B27 supplements, 4 mM Glutamax, 10 µg/mL gentamicin, and 2.50 µg/mL amphotericin B. FRDA mouse cells were maintained by replacing half of the old medium with fresh neurobasal medium/B27 every 2–3 days. At day 3–5, 5-fluoro-2′-deoxyuridine (FDU) (5 µM) (Sigma-Aldrich, St. Louis, MO, USA) was included in culture medium to inhibit the growth of glial cells. Neuronal cells were used for the experiments after being cultured for 10 days. FRDA mouse cerebellar neuronal cells were validated by detecting a cerebellar neuronal marker, Tuj1, also named as tubulin beta 3 class III, on neuronal cells, and Zic2 and CALB1 on cerebellar granule and Purkinje cells using immunofluorescence (IF) with antibodies (Tuj1 antibody, ab18207, Zic2 antibody, ab150404, and CALB1 antibody, ab108404) (Abcam, Waltham, MA, USA) against the neuronal markers according to the protocol provided by Abcam (Supplementary Figure S2A). The residual glial cells in neural culture were detected using the antibody against the BLBP glial cell marker (Abcam, ab32423, Waltham, MA, USA) (Supplementary Figure S2A) using IF. In brief, mouse FRDA neuronal cells were fixed with 100% methanol for 5 min. Cells were then permeabilized with 0.1% Tween-20 in PBS for 5 min and subjected to blocking with the solution containing 1% BSA, 0.3 M glycine, and 0.1% Tween-20 in PBS for 1 h. Cells were then incubated with the antibodies against various neuronal markers at 1 μg/mL overnight at 4 °C. Cells were washed with 0.1% Tween-20 in PBS three times and incubated with a secondary antibody to rabbit IgG-H&L (Alexa Fluor 488, green color or Alexa Fluor 594, red color) at 1:1000 dilution. Cells were stained for their nucleus with DAPI at 0.1 μg/mL. Cell images were acquired using a Leica confocal microscope at the Florida International University Confocal Microscopy Core Facility at a scale of 100 or 200 μm. Neuronal cells were treated with 10 µM of temozolomide (Sigma-Aldrich, St. Louis, MO, USA) for 2 h, followed by 2 days of recovery. The cells were repeatedly treated three times using the same scheme. Untreated FRDA cerebellar neuronal cells were used as the control. Cells were then subjected to chromatin immunoprecipitation (ChIP) or harvested to determine the instability of the expanded GAA repeats at the *FXN* gene. The experiments were performed at least two or three times with biological replicates.

### 2.6. FRDA Neural Cell Differentiation from FRDA Patient Induced Pluripotent Stem Cells (iPSCs) and Cellular Treatment by BIX01294

FRDA iPSCs were obtained from Coriell Institute for Medical Research (Cat. No. GM23404) (Camden, NJ, USA) and seeded in a Matrigel-coated 6-well plate at 1000 cells/well and cultured in neural induction media containing 1:1 Advanced DMEM/F-12:Neurobasal media supplemented with 1× N2, 1× B27, 1% Glutamax, leukemia inhibitory factor [hLIF] (MilliporeSigma, Burlington, MA, USA) (10 ng/mL) at about 20% confluence. FRDA neural cell differentiation was conducted using a protocol described previously [45]. Briefly, FRDA iPSCs were switched to a neural induction medium containing CHIR99021 (CHIR) (Cellagen Technology, San Diego, CA, USA) (3 μM), SB431542 (SB) (Cellagen Technology, San Diego, CA, USA) (2 μM), and Compound E (γ-secretase Inhibitor XXI) (MilliporeSigma, Burlington, MA, USA) (0.1 μM)] for 7 days. Cells were then split at a 1:3 ratio for six passages using Accutase (ThermoFisherScientific, Waltham, MA, USA) and cultured in a neural induction medium supplied with 3 μM CHIR, 2 μM SB, and 0.1 μM Compound E. After six passages, cells were split 1:10 regularly and differentiated into FRDA neural progenitor cells (NPCs). FRDA NPCs were plated at an ultra-low density at 200 cells/well in a 6-well plate coated by both Matrigel and poly-L-ornithine and cultured in a neural induction medium containing 3 μM CHIR, 2 μM SB, and 0.1 μM Compound E and grown for 3 days. Subsequently, FRDA NPCs were switched to a neural differentiation medium containing DMEM/F12, 1× N2, 1× B27, 300 ng/mL cAMP (Sigma-Aldrich), 0.2 mM vitamin C (Sigma-Aldrich, St. Louis, MO, USA), 10 ng/mL BDNF, and 10 ng/mL GDNF and subjected to neural differentiation for 14 days (Supplementary Figure S2B). Neuronal cells were identified by staining the cytoskeleton neuronal marker MAP2 and general neuronal marker Tuj1 in differentiated FRDA neural cells using an anti-MAP2 (Abcam, ab32454, Waltham, MA, USA) and anti-beta III tubulin (Abcam, ab18207, Waltham, MA, USA) neuronal marker antibody using IF (Supplementary Figure S2B). The cells exhibiting pluripotency were detected using an anti-OCT4 antibody (Abcam, ab19857, Waltham, MA, USA). Cells were incubated with the antibodies to the neuronal and pluripotency markers at 1 μg/mL overnight at 4 °C and washed with 0.1% Tween in PBS. Cells were then incubated with a secondary antibody to rabbit IgG-H&L (Alexa Fluor 488, green color, or Alexa Fluor 594, red color) at 1:1000 dilution. The nucleus in cells was stained using DAPI at 0.1 μg/mL. Cell images were acquired using a Leica confocal microscope at a scale of 200 μm. FRDA neural cells were treated with 0.5 μM and 1 μM BIX01294 for 72 h. Untreated cells were used as the control. FRDA neural cells were collected to determine the level of Fxn protein, contraction of the expanded GAA repeats, inhibition of H3K9 methyltransferase activity, the profiles of single-strand DNA breaks on the expanded GAA repeats, and recruitment of DNA polymerase β (pol β) with ChIP.

### 2.7. Measurement of GAA Repeat Instability at the FXN Gene

Genomic DNA was isolated from FRDA mouse brain tissue, primary cultured FRDA neuronal cells, and differentiated human FRDA neural cells using Wizard Genomic DNA Purification Kit (Promega, Madison, WI, USA). GAA repeats at the *FXN* gene were amplified with the Long Range PCR kit (New England Biolabs (Ipswich, MA, USA) using a forward primer (5′-GCC AAC ATG GTG AAA CCC AGT ATC-3′) and a reverse primer tagged by a 6-carboxyfluorescein (5′-6-FAM- CCA CGC CCG GCT AAC TTT TC-3′) as reported previously by our group [38]. The PCR program used for the amplification of GAA repeats was as follows: 94 °C for 20 s, 65 °C for 2 min (annealing and extension), 20 cycles; 94 °C for 20 s, 65 °C for 2 min in which the time of this step was increased by 15 s per cycle (annealing), 65 °C for 1.5 min (extension), 17 cycles; and final extension at 65 °C for 30 min. PCR products with various lengths of GAA repeats were generated. The amplified fluorescence (6-FAM)-labeled PCR products were then subjected to capillary electrophoresis for the separation of DNA fragments with various sizes of expanded GAA repeats at Florida International University DNA Sequencing Core. The size standard, MapMarker 2000 (300–2000 bp) (Bioventures, Murfreesboro, TN, USA), was employed for the repeat size determination. The sizes of GAA repeats were determined using DNA fragment analysis (GeneMapper version 4.0 software, Applied Biosystems, Foster City, CA, USA). The size of the DNA fragments was calculated as 79+3n, where “n” represented the repeat number. The percentage of the GAA repeat expansion and contraction products was calculated by the following equation:

$$\frac{\mathrm{The}\;\mathrm{sum}\;\mathrm{of}\;\mathrm{the}\;\mathrm{peak}\;\mathrm{height}\;\mathrm{of}\;\mathrm{expansion}\;\mathrm{or}\;\mathrm{contraction}\;\mathrm{products}}{\mathrm{The}\;\mathrm{sum}\;\mathrm{of}\;\mathrm{the}\;\mathrm{peak}\;\mathrm{height}\;\mathrm{of}\;\mathrm{all}\;\mathrm{expansion}\;\mathrm{and}\;\mathrm{contraction}\;\mathrm{products}}\times 100\%$$

### 2.8. The Profiles of Single-Strand DNA Breaks (ssDNA) on the Expanded GAA Repeats at the FXN Gene Determined by Strand Break-Mediated DNA Modification PROFILING Assay (SBDM)

TMZ-induced alkylated DNA bases and endogenously induced oxidized DNA bases and their locations on the expanded GAA repeats at the *FXN* gene of FRDA mouse neuronal cells and differentiated human FRDA neural cells were mapped by determining their corresponding ssDNA break intermediates converted by endogenous alkyladenine DNA glycosylase (AAG) or 8-oxoguanine DNA glycosylase 1 (OGG1) and AP endonuclease 1 (APE1) using the strand break-mediated DNA modification profiling assay (SBDM) (Supplementary Figure S3), also named as DNA damage landscape assay, recently developed by our group [46]. ssDNA break intermediates on GAA repeats were 5′-phosphorylated by T4 polynucleotide kinase (PNK) (10U) (ThermoFisherScientific, Waltham, MA, USA). Subsequently, primer 1 (5′-GAG TAG CTG GGA TTA CAG GC-3′) was annealed to the 3′-flanking region of the GAA repeats at the *FXN* gene and extended by Deep Vent DNA polymerase 2 U (New England Biolabs), generating double-strand DNA (dsDNA) intermediates with their sizes representing the locations of alkylated or oxidized bases. The dsDNA intermediates were then ligated to a barcode dsDNA (top strand: 5′-C TGA TGT CGG ACC ATC GAG AGT GCG-3′, bottom strand: 5′-CGC ACT CTC GAT GGT CCG AC-3′). The ligated DNA fragments with varying lengths were amplified by long-range PCR using the 6-FAM (carboxyfluorescein)-tagged barcode primer barcode primer (5′-6-FAM GAT GTC GGA CCA TCG AGA GTG CG-3′) and primer 2 (5′-CGA CAC CAC GCC CGG CTA AC-3′) that was annealed to the region closer to the GAA repeats at the *FXN* gene. The amplified fluorescence-tagged PCR products were then subjected to capillary electrophoresis (Florida International University DNA Sequencing Core Facility) and DNA fragment analysis (GeneMapper version 4.0 software, Applied Biosystems, Foster City, CA, USA) for their size determination. The size standard, MapMarker 500 (Bioventures, Murfreesboro, TN, USA), was run in parallel with PCR products. The locations of ssDNA breaks on the expanded GAA repeats were mapped according to the size of the amplified PCR products using the sizes of the fragments after subtracting the sizes of the barcode primer and primer 2. The unique profiles of base lesions were then generated (Supplementary Figure S3).

### 2.9. Histone Modifications and Recruitment of Pol β on the Expanded GAA Repeats Determined by ChIP Assay

A total of 5 × 10^4^ primary cerebellar neural cells isolated from the cerebellum tissue of 3-day-old FRDA mouse pups and 5 × 10^5^ neural cells differentiated from FRDA iPSCs were treated with 10 μM TMZ for 2 h. Primary and differentiated FRDA neural cells treated with 0.1% DMSO in saline were used as a negative control. At the end of the treatment, cells were washed twice using 1X PBS. Cells were then subjected to the experimental procedures of ChIP assay, as reported previously by our group [39]. Briefly, primary mouse FRDA neural cells and differentiated human FRDA neural cells were supplied with medium containing 1% formaldehyde and incubated at 37 °C for 30 min for a crosslinking reaction that was subsequently quenched by 125 mM glycine for 5 min with shaking. Cells were collected through cell scraping and centrifugation. Cell pellets were washed twice with cold 1 X PBS containing a mixture of protease inhibitors [39] and suspended in a cell lysis buffer with 1% SDS, 10 mM EDTA, and 50 mM Tris HCl, and protease inhibitors with a pH of 8.0. The cell suspension was then incubated on ice for 10 min and subjected to lysis by sonication with Bioruptor Ultrasonicator (Diagenode, Denville, NJ, USA) at 4 °C for 15 cycles of 30 s ON and 30 s OFF for cell lysis and DNA fragmentation. Cell lysates were subjected to centrifugation at 18,000× *g* for 10 min at 4 °C to separate the supernatant from cell debris. Subsequently, the supernatant was diluted 10-fold with ice-cold ChIP dilution buffer containing protease inhibitors. 150 µL of the supernatant was taken as the input. The remaining supernatant was incubated with sheared salmon sperm DNA-coated protein A Sepharose (Life Technologies/ThemoFisherScientific, Carlsbad, CA, USA) for 2 h at 4 °C with rotation. The supernatant was then divided into equal aliquots that were incubated with 2 µg IgG (Santa Cruz Biotechnology, sc-2025, Dallas, TX, USA) (No antibody control) or 2 µg anti-H3K9me3 antibody (Abcam, Ab8898, Waltham, MA, USA), anti-H3K9ac antibody (Abcam, ab10812, Waltham, MA, USA), and anti-mouse and human pol β antibody (Abcam, ab26343, Waltham, MA, USA), respectively. IPs of pol β, H3K9me3, H3K9ac, and IgG were incubated with sheared salmon sperm DNA-coated protein A-Sepharose beads for 2 h at 4 °C with rotation. IPs bound to the beads were precipitated by centrifugation at 500× *g* for 2 min and washed twice with low-salt washing buffer containing 150 mM NaCl, followed by washing with high-salt buffer with 500 mM NaCl, and finally washed with TE buffer. The IPs were eluted from the beads with freshly made buffer containing 1% SDS and 0.1 M NaHCO_3_. The IPs were incubated with 0.2 M NaCl at 65 °C for 6 h for DNA-protein crosslink reversal, leading to the release of the precipitated DNA from the protein. The IPs were then subjected to proteinase K digestion at 45 °C for 2 h to remove proteins. The released DNA was cleaned up with phenol/chloroform extraction, precipitated with ethanol, and redissolved in TE buffer for subsequent quantitative PCR amplification of GAA repeats at the *FXN* gene bound by pol β, H3K9me3, H3K9ac, and IgG.

### 2.10. Quantitative Real-Time PCR for ChIP Assay

The quantitative real-time PCR was performed according to the protocol reported by our group [39]. The PCR reaction was assembled with SYBR Green Supermix (Bio-Rad Laboratories), purified DNA bound by pol β, H3K9me3, H3K9ac, and IgG, and the forward and reverse PCR primers (forward primer: 5′-GGT ATT TTT GGTTGC TTA AAA G-3′ and reverse primer: 5′-CCT ATT TTT CCA GAG ATG CTG GGA AAT CC-3′) in 20 µL reaction mixture. The PCR amplification was performed using the CFX Connect Real-Time PCR Detection System (Bio-Rad Laboratories) with a PCR program as follows: 98 °C for 2 min for 1 cycle, 98 °C for 20 sec, 51 °C for 1 min, and 72 °C for 2 min for 40 cycles, leaving PCR products with 422+3n bp, where “n” represented the number of GAA repeats. Ct values recorded by the CFX Manage version 3.1 Software (Bio-Rad Laboratories, Hercules, CA, USA) during PCR amplification were used to calculate the fold-difference between the amount of DNA bound by pol β, H3K9me3, H3K9ac, IgG, and the input. The equation used was ΔCt _[normalized ChIP] (normalized to the Input)_ = Ct _[ChIP]_ − (Ct _[Input]_ − log2 [input dilution factor]), where the input dilution factor = the fraction volume used for each IP/the fraction volume of input saved for further analysis. In our experiments, the fraction volume of the input was 100 μL. The fraction volume for each IP was 350 μL. The input dilution factor was 3.5. For our experiments, the equation was ΔCt _[normalized ChIP]_ = Ct _[ChIP]_ − (Ct _[Input]_ − log2 [3.5]). The input %= 2 ^−ΔCt^ [normalized ChIP] × 100. The “input %” indicates the enrichment of H3K9me3, H3K9ac, pol β, and IgG on the expanded GAA repeats of the *FXN* gene. The data was subjected to statistical analysis using the GraphPad Prism version 6 software with two-way ANOVA. *p* < 0.05 was designated as a significant difference. The experiments were performed at least three times with biological replicates.

### 2.11. Measurement of Frataxin (Fxn) Protein Level in Mouse Brain Tissue and Differentiated Human FRDA Neural Cells Using Immunoblotting

Mouse brain tissue was homogenized and then subjected to cell lysis using cell lysis buffer containing 150 mM NaCl, 50 mM Tris HCl pH = 8.0, 1% NP-40, and protease inhibitor cocktail (Pierce Biotechnology/ThermoFisherScientific, Waltham, MA, USA). Differentiated FRDA neural cells were directly subjected to cell lysis buffer with a protease inhibitor cocktail. Tissue and cell lysates were then subjected to centrifugation at 12,000 rpm for 30 min at 4 °C. The supernatant of tissue and cell lysates was collected, and their protein concentrations were determined using the Bradford assay. Total protein of 30 μg of each sample was subjected to SDS-PAGE and immunoblotting with a mouse anti-Fxn antibody (1:1000, Abcam ab110328, Waltham, MA, USA) and a mouse anti-β-actin antibody (1:1000; Abcam ab8226 for mouse and human β-actin), followed by incubating with rabbit anti-mouse IgG H&L (HRP) (1:7000; ab6728, Abcam, Waltham, MA, USA). Proteins were then detected by chemiluminescence using Pierce ECL Western blotting substrate (Pierce Biotechnology/ThermoFisher Scientific, Waltham, MA, USA). The experiments were performed in biological triplicate.

### 2.12. Measurement of the Inhibition of Nuclear H3K9 Methyltransferase Activities of Human FRDA Neural Cells by TMZ and BIX01294 Using EpiQuik™ Histone Methyltransferase Activity/Inhibition Assay Kit (H3-K9)

The effects of TMZ and BIX01294 on the activities of H3K9 methyltransferases in differentiated human FRDA neural cells were determined using EpiQuik™ histone methyltransferase activity/Inhibition assay kit (H3-K9) (EpigenTek, Farmingdale, NY, USA). The nuclear extract of FRDA neural cells was made using the nuclear fractionation protocol from Abcam (Waltham, MA, USA). Briefly, cells were harvested into 500 μL buffer A (10 mM HEPES, pH 7.9, 1.5 mM MgCl_2_, 10 mM KCl, 0.5 mM DTT, and 0.05% NP-40) and incubated on ice for 10 min to release cytoplasm. Cells were then subjected to centrifugation at 3000 rpm for 10 min at 4 °C, allowing for the precipitation of the nucleus. The supernatant was then removed, and the nucleus pellets were resuspended using buffer B [5 mM HEPES, pH 7.9, 300 mM NaCl, 1.5 mM MgCl_2_, 0.2 mM EDTA, 0.5 mM DTT, 26% glycerol (*v*/*v*)]. The nucleus was homogenized using a glass homogenizer with 20 full strokes on ice. Nuclear lysates were incubated on ice for 30 min and subjected to centrifugation at 12,000 rpm for 20 min at 4 °C. The supernatant, i.e., nuclear extract, was aliquoted and stored at −80 °C for the H3K9 methyltransferase assay. FRDA neural nuclear extract (20 μg) was incubated with 10 μM TMZ or 1 μM BIX01294 at 4 °C overnight with rotation. The nuclear extract was then incubated with the substrate provided by the EpiQuik™ histone methyltransferase activity/Inhibition assay kit (H3-K9) (EpigenTek, Farmingdale, NY, USA) for 1 h and subjected to H3K9 methyltransferase activity measurement according to the protocol provided by EpigenTek (Farmingdale, NY, USA). The absorbance at 450 nm of the samples was obtained using a BioTek plate reader (BioTek/Agilent Technologies, Santa Clara, CA, USA). H3K9 methyltransferase activity was calculated according to the equations provided by the manufacturer.

$$\mathrm{Inhibition}\;\left(\%\right)=(1-\frac{\mathrm{OD}\;\left(\mathrm{inhibitor}\;\mathrm{sample}-\mathrm{blank}\right)}{\mathrm{OD}\;\left(\mathrm{no}\;\mathrm{inhibitor}\;\mathrm{control}-\mathrm{blank}\right)})\times 100\%$$

### 2.13. Data and Statistical Analysis

All the results were obtained from at least two or three biological replicates. The bar charts were created using the GraphPad Prism version 6 software. Statistical analysis was conducted using a one or two-way ANOVA and student *t*-test with the GraphPad Prism version 6 software.

## 3. Results

### 3.1. TMZ Preferentially Induces Contractions of Expanded GAA Repeats and Increases Fxn Protein Levels in FRDA Transgenic Mouse Brain Tissue

**Figure 1:**
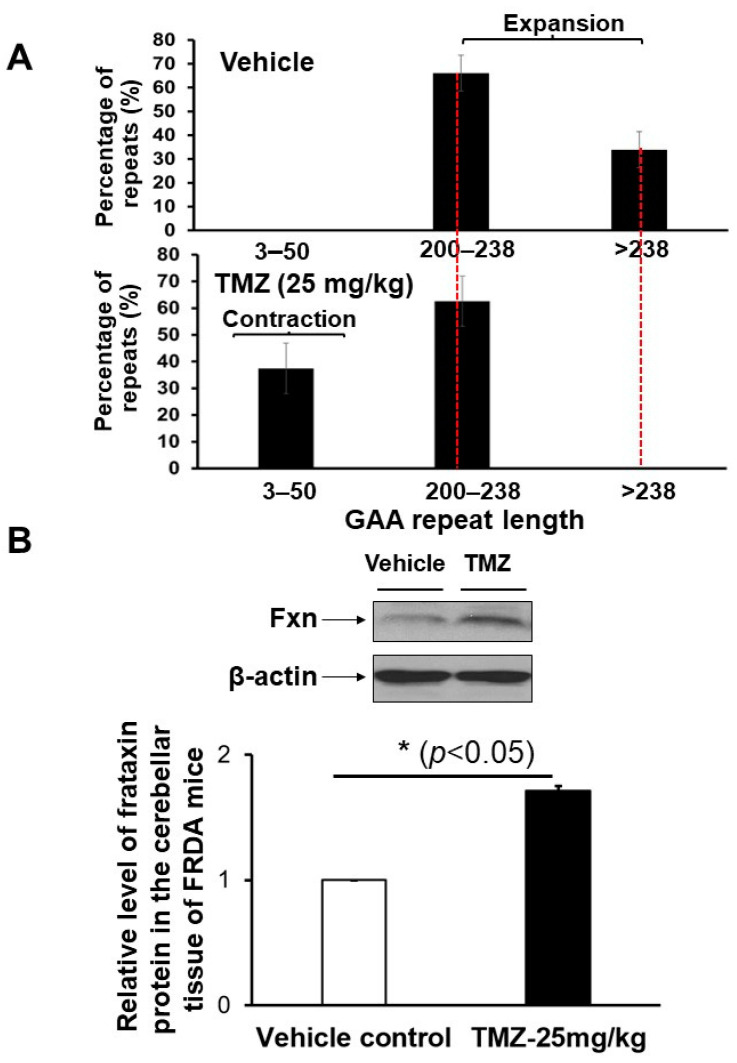
TMZ treatment induces GAA repeat contraction, leading to increased Fxn protein levels in FRDA mouse brain tissue. FRDA transgenic mice (12 weeks old, male) were treated with 25 mg/kg TMZ three times per week for 3 months through oral administration. (**A**) The effect of TMZ on the large contraction of the expanded GAA repeats in the brain tissue of FRDA mice was determined using capillary electrophoresis and DNA fragment analysis. The percentage of GAA repeat-containing fragments with specific repeat size ranges among the total amount of all repeat-containing fragments was calculated. (**B**) The TMZ effects on the level of Fxn protein of FRDA mouse brain and its quantification were shown. Fxn protein levels in mouse brain tissue were detected using immunoblotting with an anti-human Fxn antibody. Mouse β-actin was used as the loading control and detected with an anti-mouse/human β-actin antibody. Representative gel images were shown. The results were obtained from two biological replicates. The original gel images were included in Figure S4.

Initially, we asked if TMZ can preferentially induce contractions of expanded GAA repeats in FRDA mouse brain tissue. FRDA transgenic mice, aged three months, were treated with 25 mg/kg TMZ three times per week over three months through oral gavage. The mouse brain tissue was harvested and subjected to the determination of GAA repeat length change at the *FXN* gene and Fxn protein level (Figure 1). The results showed that in the control mouse brain tissue, the expanded GAA repeats ranged from 200 to larger than 238 repeats, with 66% of expansion products having 200–238 repeats and 34% of them containing repeats longer than 238 repeat units (Figure 1A). TMZ treatment at 25 mg/kg resulted in the disappearance of repeat products larger than 238 repeats, leading to 63% of expansion products with 200–238 repeats (Figure 1A, the bottom panel). Furthermore, TMZ treatment led to 37% of contraction products containing 3–50 repeat contraction products (Figure 1A, the bottom panel). The results indicate that TMZ treatment led to a substantial contraction of expanded GAA repeats at the *FXN* gene in FRDA transgenic mouse brain tissue (Figure 1A, the bottom panel). To further determine if TMZ could upregulate *FXN* gene expression, the Fxn protein levels in FRDA mouse brain tissue upon TMZ treatment were measured. The results showed that TMZ increased the Fxn protein levels in FRDA mouse brain tissue by 1.7-fold (*p* < 0.05) (Figure 1B). The results further indicated that TMZ induced the contraction of the expanded GAA repeats at the *FXN* gene in the FRDA mouse brain, leading to the upregulation of the *FXN* gene.

### 3.2. TMZ Can Induce the Contraction of the Expanded GAA Repeats by Reducing H3K9 Trimethylation (H3K9me3) and Facilitating BER of ssDNA Breaks on the Repeats in FRDA Transgenic Mouse Neuronal Cells

**Figure 2:**
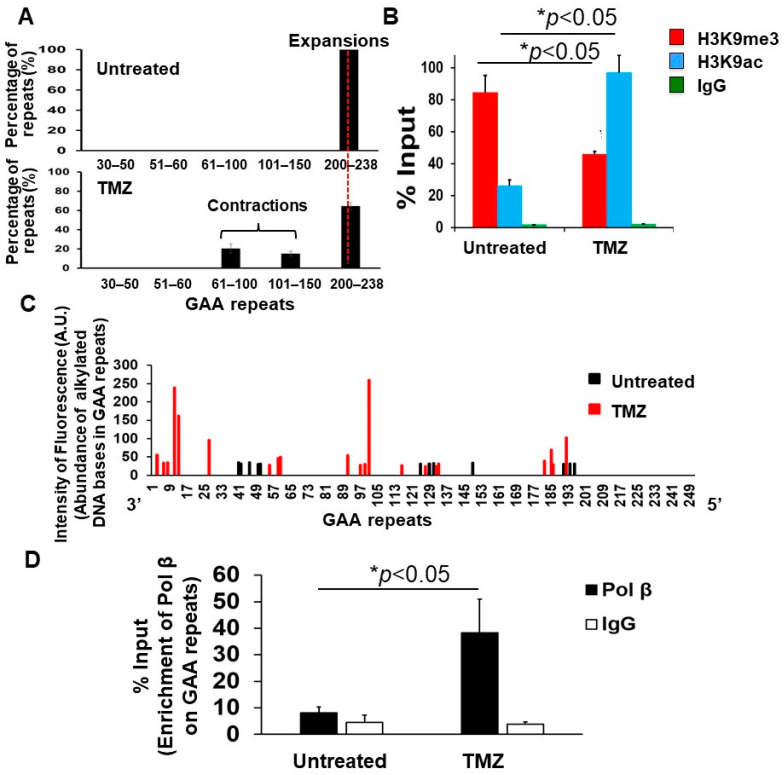
TMZ leads to the opening of the chromatin on the expanded GAA repeats at the *FXN* gene, promoting the accumulation of ssDNA breaks and the recruitment of Pol β on the repeats in FRDA transgenic mouse cerebellar neuronal cells. (**A**) The effects of TMZ on the contraction of the expanded GAA repeats at the *FXN* gene in FRDA transgenic mouse cerebellar neuronal cells were determined using capillary electrophoresis and DNA fragment analysis. The percentage of GAA repeat-containing fragments with specific repeat size ranges among the total amount of all repeat-containing fragments was calculated. Cells were treated with 10 μM TMZ for 24 h and recovered for 48 h repeatedly three times. (**B**) The effects of TMZ on histone H3K9me3 and H3K9ac at the *FXN* gene in FRDA mouse cerebellar neuronal cells were detected using the ChIP assay with an anti-H3K9me3 and anti-H3K9ac antibody, respectively. Cells were treated with 10 μM TMZ for 2 h. The results were obtained from three biological replicates. (**C**) TMZ-induced accumulation of ssDNA breaks on the expanded GAA repeats at the *FXN* gene in FRDA transgenic mouse cerebellar neuronal cells was detected using the SBDM assay (Supplementary Figure S3). The black and red bars represent the ssDNA on the GAA repeats from untreated and TMZ-treated cells. Cells were treated with 10 μM TMZ for 2 h and harvested. ssDNA breaks resulting from alkylated DNA bases induced by TMZ and endogenous oxidative DNA base damage were mapped on GAA repeats. (**D**) The recruitment of pol β to the expanded GAA repeats at the *FXN* gene in FRDA transgenic mouse cerebellar neuronal cells was determined with the ChIP assay using anti-mouse and human pol β antibody. Cells were treated with 10 μM TMZ for 2 h. Untreated cells were used as the control. The experiments were repeated at least two or three times with biological replicates.

Since TMZ can induce alkylated DNA bases that are subjected to the BER pathway [47], we then ask if TMZ can cause GAA repeat contraction in FRDA transgenic mice by increasing the accumulation of ssDNA breaks resulting from alkylated DNA bases and endogenously-induced oxidized DNA bases on the expanded GAA repeats at the *FXN* gene and if this can subsequently result in the opening of the chromatin and recruitment of BER enzymes that repair the ssDNA breaks on the expanded repeats, leading to repeat contraction. To test this, cerebellar neuronal cells isolated from 3-day-old FRDA mouse cerebellum were employed to determine the effects of TMZ on the length of the expanded GAA repeats, histone modifications H3K9me3 and H3K9 acetylation (H3K9ac), the accumulation of ssDNA breaks, and the recruitment of the key BER enzyme, DNA polymerase β (pol β), on the expanded repeats (Figure 2). We found that TMZ at 10 µM also resulted in the contraction of the expanded GAA repeats in FRDA mouse cerebellar neuronal cells by converting ~35% of the expansion products with 200–238 repeats into contraction products containing 61–100 and 101–150 repeats (Figure 2A). The results are consistent with the effects of TMZ on GAA repeat contraction in FRDA mouse brain tissue. Using the ChIP assay, TMZ was found to significantly reduce H3K9me3 and increase H3K9ac in FRDA mouse cerebellar neuronal cells (Figure 2B). Using the SBDM profiling assay [46] (Supplementary Figure S3), we further demonstrated that TMZ significantly increased the accumulation of ssDNA breaks on the expanded GAA repeats that were evenly distributed from the 3′- to 5′-end of the expanded repeats in the neuronal cells (Figure 2C). To examine if the ssDNA breaks can recruit the key BER enzyme, pol β, onto the expanded GAA repeats, a ChIP assay was performed. The results showed that TMZ treatment increased the pol β enrichment on the expanded GAA repeats in FRDA mouse cerebellar neuronal cells by about 4-fold (Figure 2D). Our results support our hypothesis that TMZ induced the opening of the chromatin on the GAA repeats and the accumulation of ssDNA breaks on the repeats that subsequently recruited BER enzymes to activate the BER pathway, thereby leading to the contraction of the expanded repeats in FRDA neural cells.

### 3.3. TMZ Exhibits a Comparable Inhibitory Effect on H3K9 Methyltransferase Activity of FRDA Neural Cells, and Inhibition of H3K9 Methylation by BIX01294 Leads to Large Contractions of Expanded GAA Repeats in Differentiated Human FRDA Neural Cells

**Figure 3:**
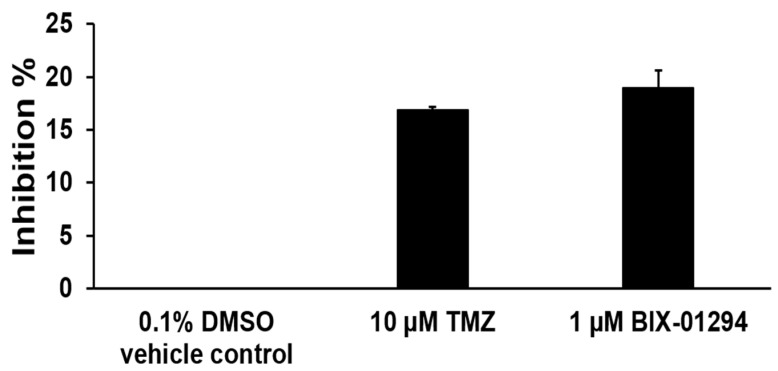
TMZ inhibits H3K9 methyltransferase activity in differentiated human FRDA neural cells. The inhibition of TMZ on H3K9 methyltransferase activity in human FRDA neural cells was measured using the EpiQuik histone methyltransferase activity/inhibition assay kit. TMZ (10 μM) and BIX01294 (1 μM) were preincubated with human FRDA nuclear extract (20 μg) overnight at 4 °C with rotation and then subjected to H3K9 methyltransferase activity assay. The inhibitory effect of BIX01294 on H3K9 methyltransferase activity was used as the positive control. DMSO (0.1%) was used as the negative control. The percentage of inhibition of TMZ and BIX01294 on H3K9 methyltransferases was calculated. The experiment was performed in triplicate.

**Figure 4:**
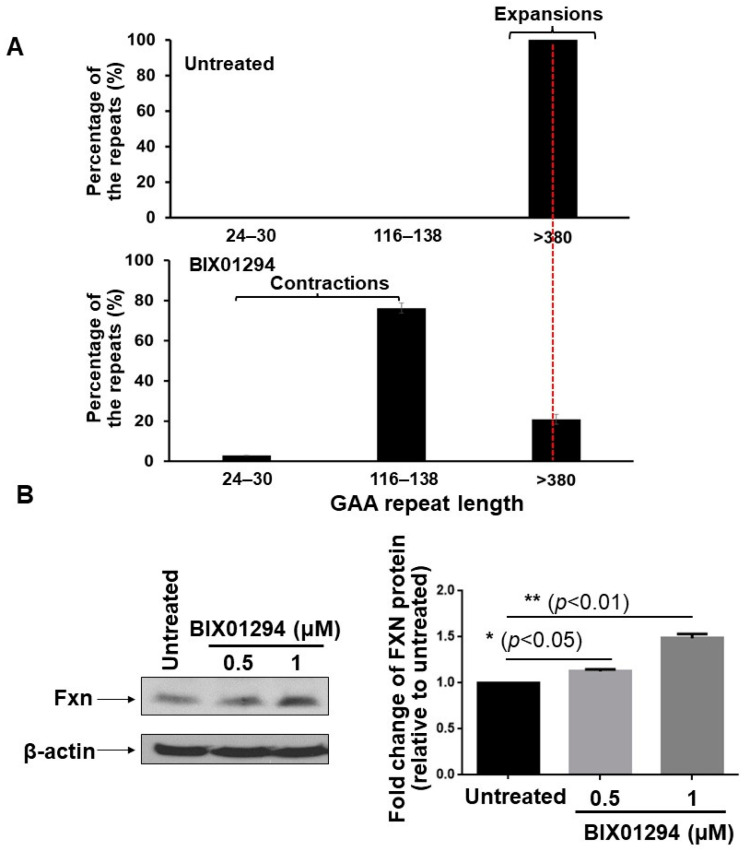
BIX01294 modulates the instability of the expanded GAA repeats at the *FXN* gene and level of Fxn protein in human FRDA neural cells. (**A**) The effects of BIX01294 on the instability of the expanded GAA repeats at the *FXN* gene in differentiated human FRDA neural cells were determined using capillary electrophoresis and DNA fragment analysis. The percentage of GAA repeats with different size ranges among the total amount (intensity) of all repeat-containing fragments was calculated. Cells were treated with 1 µM BIX01294 for 72 h. Untreated cells were used as the control. (**B**) BIX01294 effects on the Fxn protein levels in differentiated human FRDA neural cells were determined using immunoblotting with an anti-human Fxn antibody. Cells were treated with 0.5–1 µM BIX01294 for 72 h. Untreated cells were used as the control. The cellular level of β-actin was used as the loading control and detected with an anti-human and mouse β-actin antibody (the panel on the left). Representative gel images were shown. The fold-change of Fxn protein levels in FRDA neural cells upon the treatment of various concentrations of BIX01294 was quantified (the panel on the right). “*” and “**” indicate statistical significance at *p* < 0.05 and *p* < 0.01, respectively. The experiments were performed in biological duplicate.

**Figure 5:**
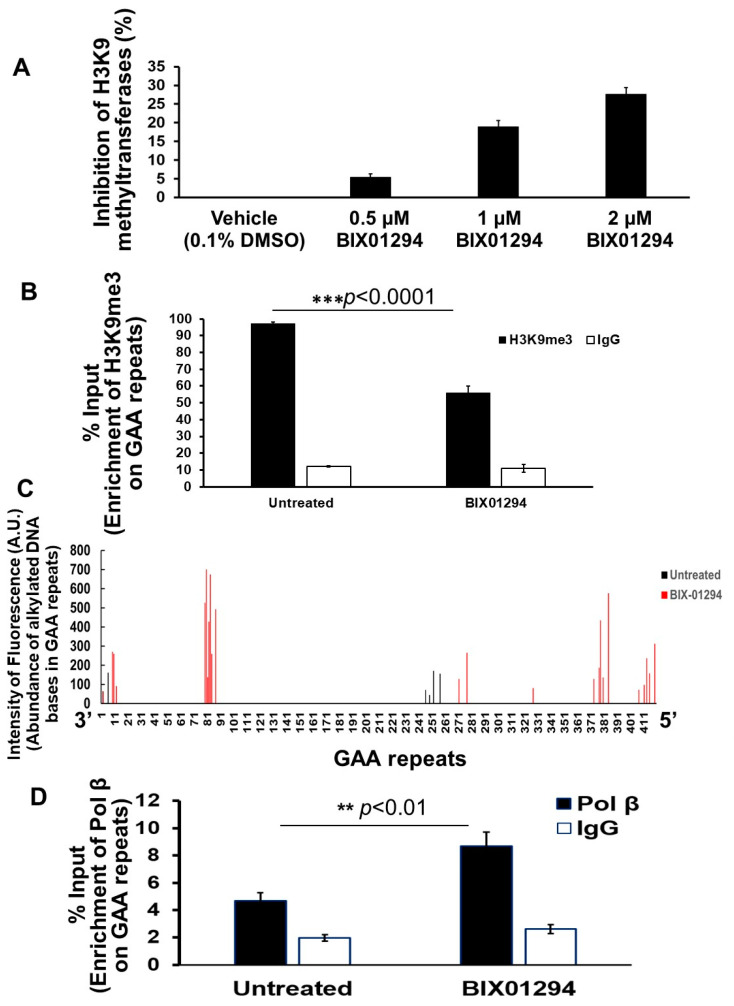
Inhibition of H3K9 methyltransferase activity by BIX01294 significantly reduces H3K9me3 on the expanded GAA repeats at the *FXN* gene in differentiated human FRDA neural cells. (**A**) The inhibitory effects of BIX01294 on H3K9 methyltransferases of FRDA neural cells at different concentrations (0.5–2 μM) were measured by preincubating the compound with human FRDA neural cell nuclear extract overnight at 4 °C. The cell nuclear extracts were then subjected to H3K9 methyltransferase assay using the EpiQuik histone methyltransferase activity/inhibition assay kit. DMSO at 0.1% was used as the control. The experiments were performed in biological triplicate. (**B**) The effects of BIX01294 on H3K9me3 on the expanded GAA repeats at the *FXN* gene in human differentiated FRDA neural cells were determined using the ChIP assay with an anti-H3K9me3 antibody. Cells were treated with 1 μM BIX01294 for 2 h and subjected to ChIP assay. IgG was used as the negative control. The experiments were performed in biological triplicate. “***” indicates a statistical significance with *p* < 0.0001. (**C**) The effects of BIX01294 on the accumulation of ssDNA breaks on the expanded GAA repeats in differentiated human FRDA neural cells were determined using the SBDM assay illustrated in Supplementary Figure S3. The green and red bars represent ssDNA on the GAA repeats in untreated and BIX01294-treated cells. Cells were treated with 1 µM BIX01294 for 24 h and harvested. ssDNA breaks on different GAA repeats were mapped. (**D**) The recruitment of pol β on the expanded GAA repeats at the *FXN* gene upon BIX01294 treatment was determined using ChIP with an anti-mouse and human pol β antibody. Cells were treated with 1 µM BIX01294 for 2 h and then subjected to ChIP assay. “**” indicates statistical significance at *p* < 0.01. IgG was used as the negative control. The experiments were performed in three biological replicates.

Since TMZ resulted in the reduction of H3K9me3, we further asked if TMZ can inhibit the activity of H3K9 methyltransferases. To test this, the activity of H3K9 methyltransferase in FRDA neural cells differentiated from FRDA patient iPSCs was measured (Figure 3). The results showed that 10 µM TMZ inhibited the H3K9 methyltransferase activity by about 17% (Figure 3). The effect was comparable with the effects from the histone methyltransferase inhibitor BIX01294 at 1 µM (Figure 3). The results further suggest that TMZ resulted in the reduction of H3K9me3 by inhibiting H3K9 histone methyltransferases in FRDA neural cells. The results allowed us to further hypothesize that the inhibition of H3K9 methyltransferases by BIX01294 can lead to the contraction of the expanded GAA repeats at the *FXN* gene. To test the hypothesis, the effects of the inhibitor of the H3K9 methyltransferases BIX01294 on the instability of the GAA repeats, and Fxn protein levels in FRDA neural cells differentiated from FRDA patient iPSCs were examined (Figure 4). The results showed that FRDA neural cells treated by 1 μM BIX01294 exhibited large GAA repeat contraction (Figure 4A, the bottom panel). About 79% of expanded repeats (larger than 380 repeats) (Figure 4A, the top panel) were contracted into the contraction products with 24–138 repeats (Figure 4A, the bottom panel). In consistency with the results, BIX01294 at 0.5 μM and 1 μM significantly increased the Fxn protein levels by 1.2–1.5-fold (*p* < 0.05) in FRDA neural cells (Figure 4B). We then validated the inhibitory effects of BIX01294 on H3K9 methyltransferase activity and demonstrated that BIX01294 at 0.5 μM, 1 μM, and 2 μM exhibited ~5%, 15%, and 25% inhibition of the H3K9 methyltransferase activity of FRDA neural cells (Figure 5A). Consistent with the results, 1 μM BIX01294 significantly reduced H3K9me3 on the expanded GAA repeats in FRDA neural cells (*p* < 0.0001) (Figure 5B), resulting in a significant increase in the amount of ssDNA breaks that spread out the expanded repeats (Figure 5C) as compared with a smaller amount of ssDNA breaks in the untreated cells localized at the 3′-end and in the middle of the repeats (Figure 5C). Furthermore, the same concentration of BIX01294 significantly increased the recruitment of pol β to the expanded GAA repeats in FRDA neural cells by ~2-fold (Figure 5D). The results indicated that the inhibition of H3K9me3 by TMZ and BIX01294 led to chromatin opening on the expanded GAA repeats, causing the accumulation of DNA damage that, in turn, recruited the key BER enzyme, pol β, to the repeats for DNA base damage repair.

## 4. Discussion

**Figure 6:**
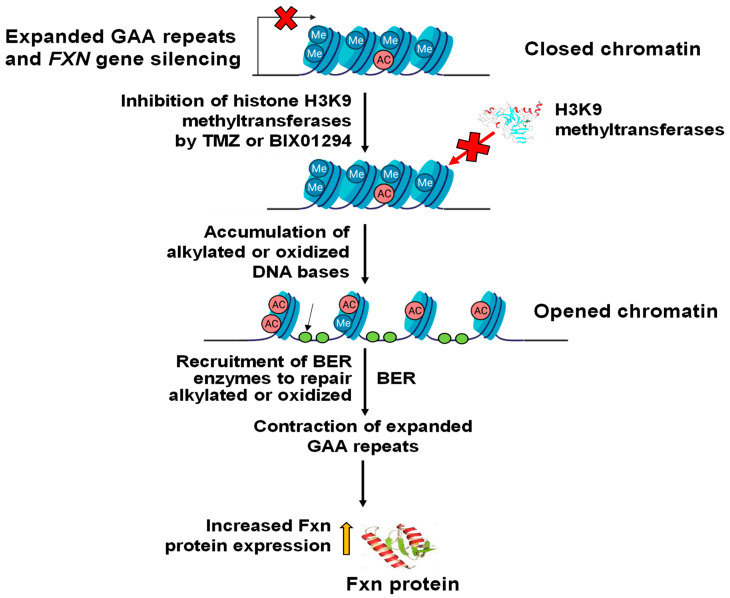
Inhibition of histone methylation synergizes with BER to contract the expanded GAA repeats. TMZ or BIX01294 inhibits H3K9 methyltransferases, leading to the opening of the chromatin on the expanded GAA repeats at the *FXN* gene in FRDA neural cells. Subsequently, this facilitates the accumulation of alkylated DNA bases and oxidized DNA bases on the expanded repeats. This, in turn, recruits BER enzymes such as methylpurine DNA glycosylase (MPG) and 8-oxoguanine DNA glycosylase 1 (OGG1), which can remove alkylated and oxidized DNA bases, initiating the BER pathway through which the expanded GAA repeats are contracted. This then increases the expression of Fxn protein in FRDA neural cells and tissues.

In this study, we provide the first evidence that TMZ results in the large contraction of expanded GAA repeats and a significant increase in Fxn protein levels in FRDA transgenic mouse brain tissue (Figure 1). We further demonstrated that TMZ also induced large GAA repeat contracts in FRDA mouse cerebellar neural cells (Figure 2A). We found that TMZ treatment decreased H3K9me3 on the expanded GAA repeats by 2-fold and increased H3K9ac by 4-fold (Figure 2B). Subsequently, this resulted in a significant increase in ssDNA breaks on the expanded GAA repeats and recruitment of pol β onto the expanded repeats (Figure 2C,D). Surprisingly, we found that TMZ also exhibited comparable inhibition of H3K9 methyltransferase activity with the enzyme inhibitor BIX-01294 in FRDA neural cell extracts (Figure 3). The results suggest that TMZ inhibited H3K9me3 and stimulated H3K9ac, leading to the disruption of heterochromatin and the opening of the chromatin on the expanded GAA repeats. Since TMZ predominantly methylates Gs and As [47,48], this then significantly increased the accessibility of TMZ to Gs and As on the expanded GAA repeats, resulting in the accumulation of methylated DNA bases N^7^-methylGs (N^7^mGs) and N^3^-methylAs (N^3^mAs) that were repaired by the BER pathway [47]. This notion was further supported by our results showing that TMZ treatment also significantly increased the recruitment of the key BER enzymes, such as pol β, on the expanded repeats (Figure 2D). We further suggest that inhibition of H3K9 methylation on the expanded GAA repeats in FRDA neural cells can lead to the opening of chromatin, increasing the accumulation of oxidized DNA bases and ssDNA breaks on the expanded GAA repeats resulting from reactive oxygen species induced by massive oxidative stress in FRDA cells [49]. Subsequently, this can initiate the BER pathway through which the expanded GAA repeats are contracted. This notion was supported by our results showing that the histone methyltransferase inhibitor BIX-01294 that inhibited H3K9 methylation in FRDA neural cell extracts (Figure 4A) and on the expanded repeats at the *FXN* gene (Figure 4B) resulted in large GAA repeat contraction in FRDA neural cells (Figure 5A) and increased Fxn protein levels (Figure 5B). Furthermore, we showed that BIX-01294 treatment also significantly increased the single-strand DNA breaks that spread out on the expanded GAA repeats (Figure 5C), resulting in the recruitment of pol β (Figure 5D). Our results support the hypothetical model where the inhibition of histone H3K9 methyltransferases by TMZ or BIX-01294 disrupts the heterochromatin on the expanded GAA repeats, opening the chromatin (Figure 6). Subsequently, this induces the accumulation of TMZ-induced alkylated DNA bases or endogenously generated oxidative DNA base damage caused by oxidative stress on the expanded repeats in FRDA brain tissue and neural cells. The DNA base damage then recruits the BER enzymes to initiate the DNA repair pathway that removes the DNA damage on the GAA repeats, during which the expanded repeats are contracted. TMZ- and BIX-01294-induced GAA repeat contraction further results in a significant increase in the Fxn protein levels in FRDA neural cells and tissues (Figure 6). Our results have proved a novel concept that the crosstalk between BER and inhibition of histone methylation will result in the large contraction of the expanded GAA repeats. Our study will open a new avenue for developing an effective treatment for FRDA through *FXN* gene-targeted GAA repeat contraction via the interplay of BER with the inhibition of histone methylation.

Our results suggest that the BER pathway can contract the expanded trinucleotide repeats in FRDA and other repeat expansion diseases through its interplay with open chromatin. This notion is also supported by a recent study showing that the CRISPR/dCas9-ten-eleven translocation 1 (TET1) fusion protein-mediated fragile mental retardation 1 (*FMR1*) gene-targeted DNA demethylation can contract the expanded CGG repeats in fragile X syndrome (FXS) iPSCs and differentiated neurons by inducing open chromatin on the expanded CGG repeats [50]. The study provided evidence that the mismatch repair protein, MSH2, can be recruited to the R-loops generated on the expanded repeats, leading to CGG repeat contraction [50]. However, it is possible that the BER pathway may also be actively involved in mediating repeat contraction based on the fact that R-loops form hotspots of DNA damage, such as ssDNA breaks that are subjected to the BER pathway, especially in neurodegenerative diseases [51]. Our recent findings show that BER in the context of R-loops without nucleosomes can preferentially lead to repeat contraction [52]. A previous study has shown that DNA repair proteins such as mismatch repair protein MSH6 can interact with H3K36me3 using the PWWP domain [53]. Further genome-wide analysis has indicated that H3K36me3-MSH6 interaction prevents mutations on actively transcribed genes induced by oxidative DNA base damage [54]. These findings suggest an interplay between H3K36me3-MSH6 interaction and the BER pathway that can facilitate BER of oxidative DNA base damage. Thus, it is conceivable that TMZ-mediated inhibition of H3K9me3 at the *FXN* gene also increases the activation histone markers such as H3K36me3 that interact with MSH6-MSH2 to stimulate BER of oxidative DNA base damage in expanded GAA repeats, leading to large repeat contraction in FRDA neural cells and brain tissue. The important roles of the interplay of BER enzymes with activation histone marks and mismatch repair proteins in modulating the expanded GAA repeat instability need to be further elucidated. Here, using TMZ as a model DNA base damaging agent, we have established a novel concept for the contraction of expanded trinucleotide repeats through the interplay between histone epigenetics, DNA base damage, and BER.

It should be noted that similar to other small molecule DNA base modifiers, TMZ can also induce alkylated Gs and As and recruitment of pol β and other BER enzymes for base damage repair in a genome-wide manner in FRDA cells and tissues. Since the expanded GAA repeats at the *FXN* gene only contain Gs and As, the repeats can form hotspots for TMZ-induced alkylated bases and BER, leading to repeat contraction at the *FXN* gene. It is expected that BER can efficiently repair TMZ-induced alkylated DNA base damage on the random DNA sequences in the genome to maintain their stability. Also, since TMZ can readily cause genome-wide mutations in brain tumor cells [55] and may exhibit global inhibition of H3K9 methyltransferases to disrupt normal neural cell function, TMZ may not be used as a treatment for FRDA. However, by studying the mechanisms underlying TMZ-induced GAA repeat contractions in FRDA, we have made the novel discovery that inhibition of histone methylation can interplay with BER to contract the expanded GAA repeats at the *FXN* gene. This will allow us to further develop an *FXN* gene-targeted inhibition of histone methylation and the recruitment of BER for effective FRDA treatment in the future.

## 5. Conclusions

Our study has demonstrated that the role of the BER pathway in maintaining genomic stability is paramount in the prevention of neurodegenerative disorders such as FRDA, thereby shedding light on the critical role of the BER pathway in relieving the pathological phenotypes of FRDA. Our findings further highlight the interplay between the inhibition of histone methylation and BER in inducing large contractions of expanded GAA repeats and upregulating *FXN* gene expression in FRDA neural cells and FRDA mouse tissues. Our study has created a platform to further understand the molecular mechanisms underlying the BER-mediated modulation of FRDA progression and opened a new avenue for developing gene therapies for FRDA and other repeat expansion diseases in the future. Our research will open a new avenue for the development of an effective treatment for FRDA through the BER pathway using an *FXN* gene-targeted approach in the future. In addition, our new concept of BER-mediated therapy for repeat expansion diseases needs to be further tested in cell and animal models of other repeat expansion diseases, such as Huntington’s disease.

## Data Availability

All the data will be deposited into a publicly accessible repository Zenodo at 10.5281/zenodo.12669729.

## Supplementary Materials

The following supporting information can be downloaded at https://www.mdpi.com/article/10.3390/biom14070809/s1, Figure S1: Schematic diagram for experiments using FRDA transgenic mice; Figure S2: Identification of primary cultured cerebellar neuronal cells from FRDA transgenic mice and differentiated human FRDA neural cells; Figure S3: Strand break-mediated DNA modification (SBDM) profiling assay for detecting single-strand breaks on GAA repeats at the FXN gene; Figure S4: Original immunoblotting images.
