# Supplementary material for: DNA Base Damage Repair Crosstalks with Chromatin Structures to Contract Expanded GAA Repeats in Friedreich’s Ataxia
Source: Biomolecules. 2024 Jul 8;14(7):809. doi: 10.3390/biom14070809 (PMC11274795; doi:10.3390/biom14070809)
Supplement: Supplementary file 1 [file biomolecules-14-00809-s001.zip › biomolecules-3066604-supplementary.pdf]

Supplementary Figure S1

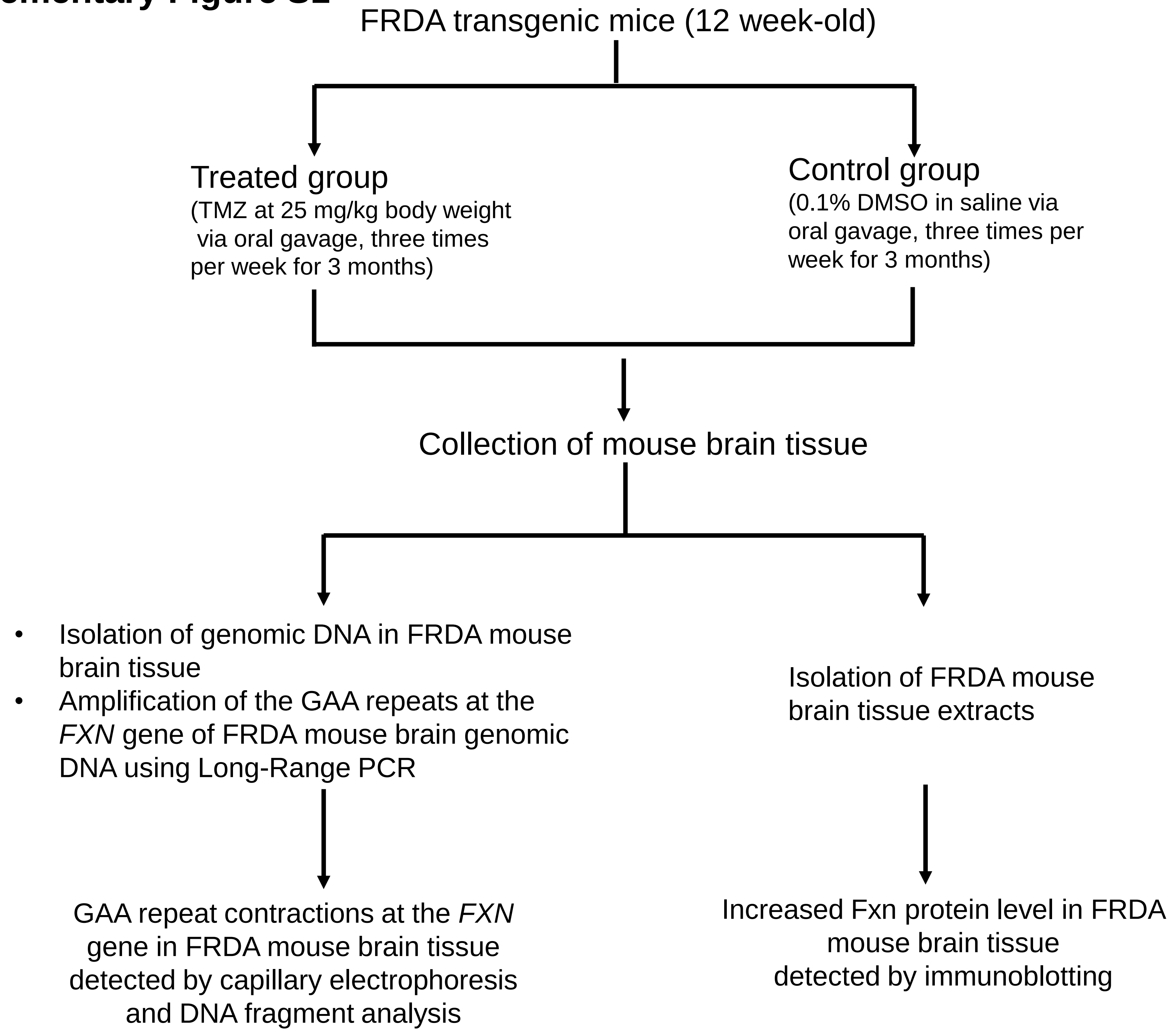

Supplementary Figure S2

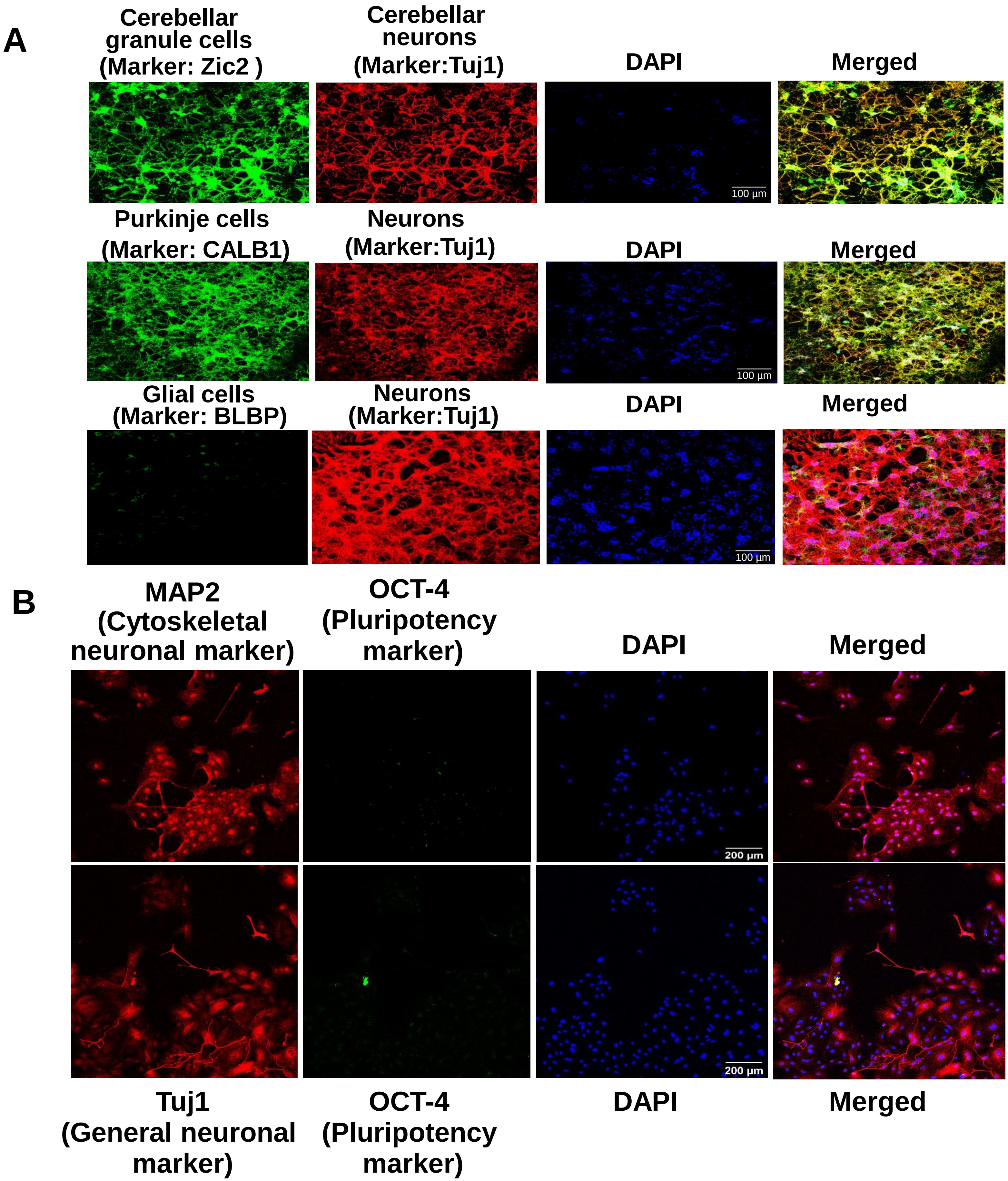

Supplementary Figure S3

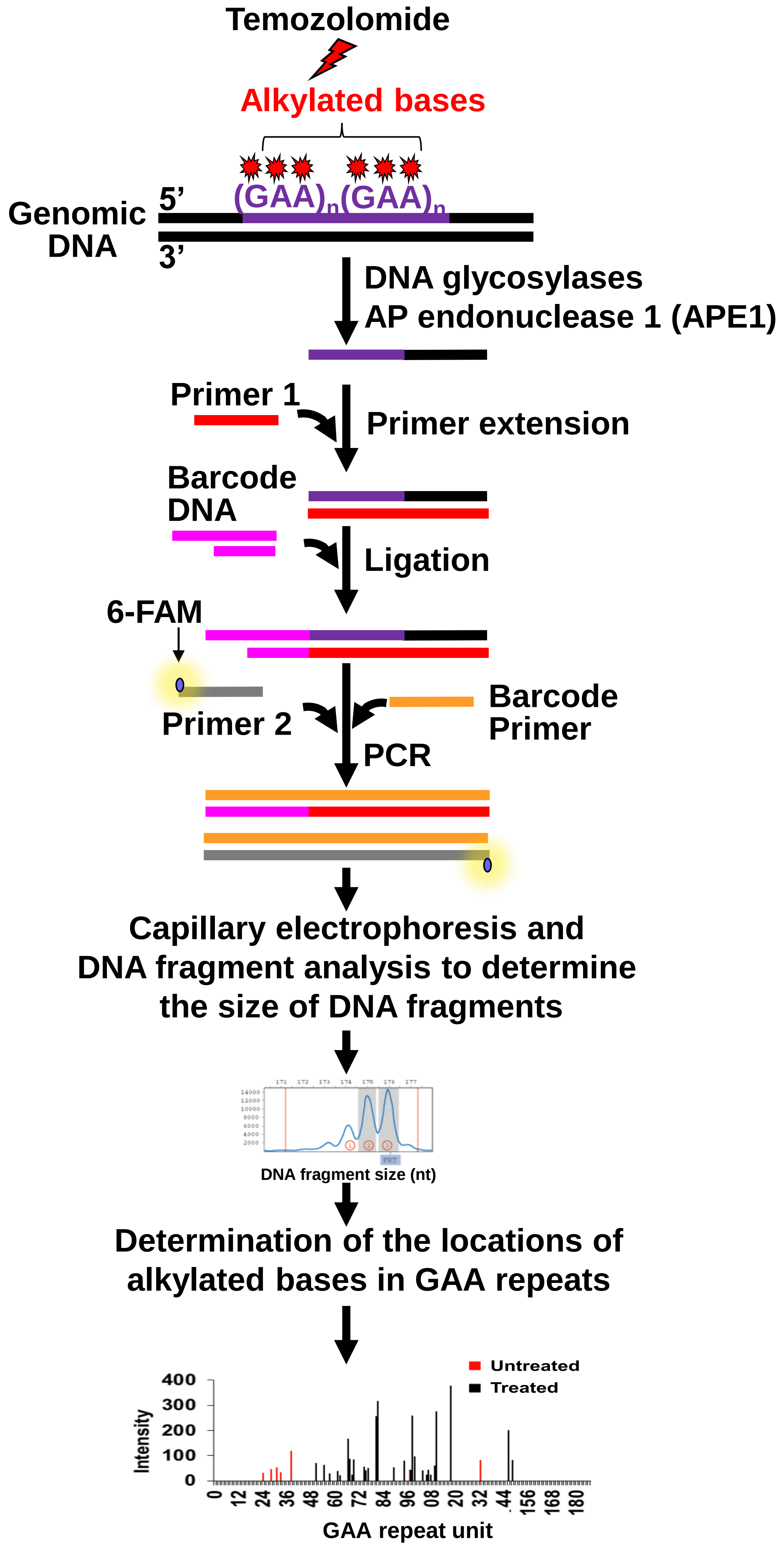

Supplementary Figure S4

A

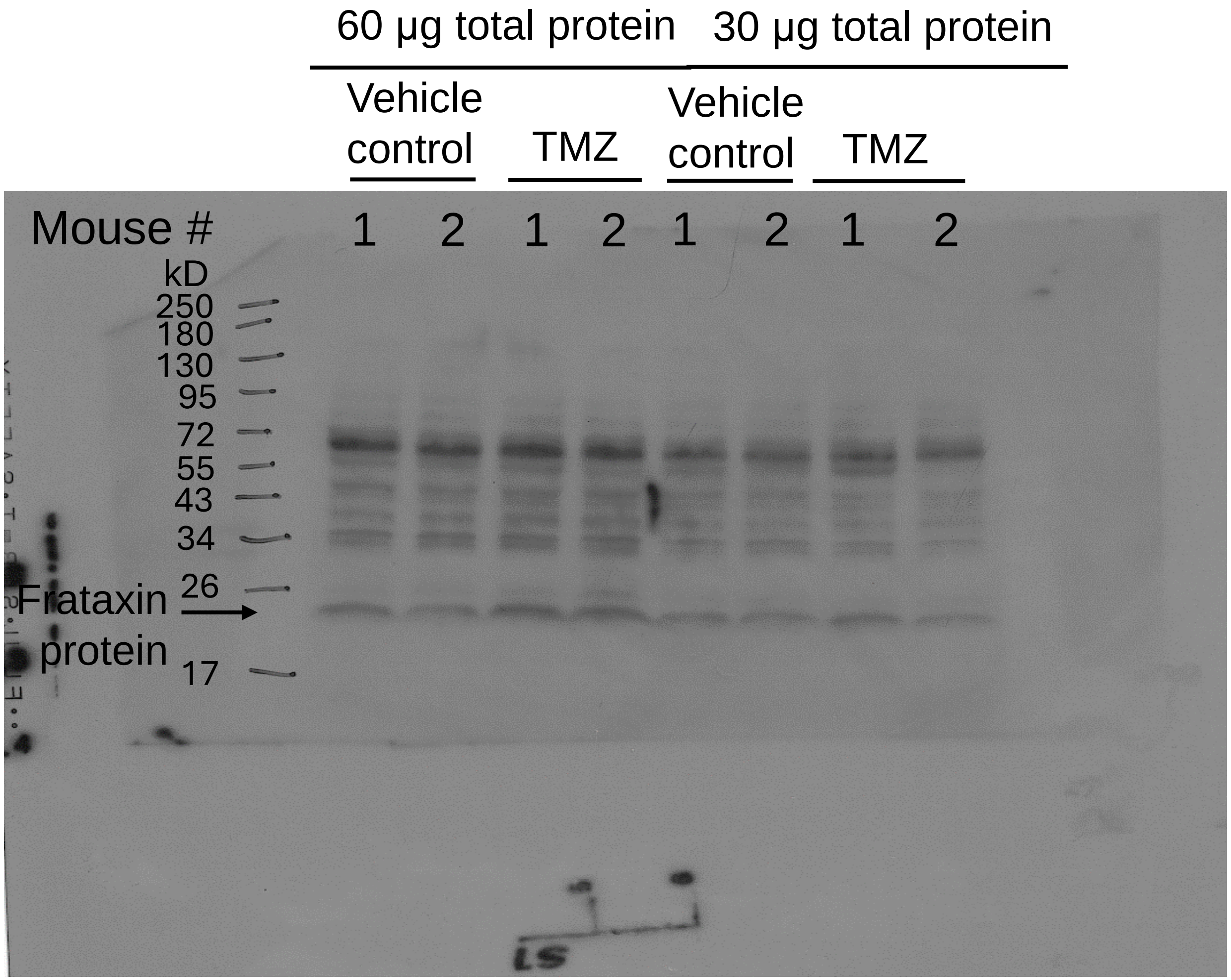

B

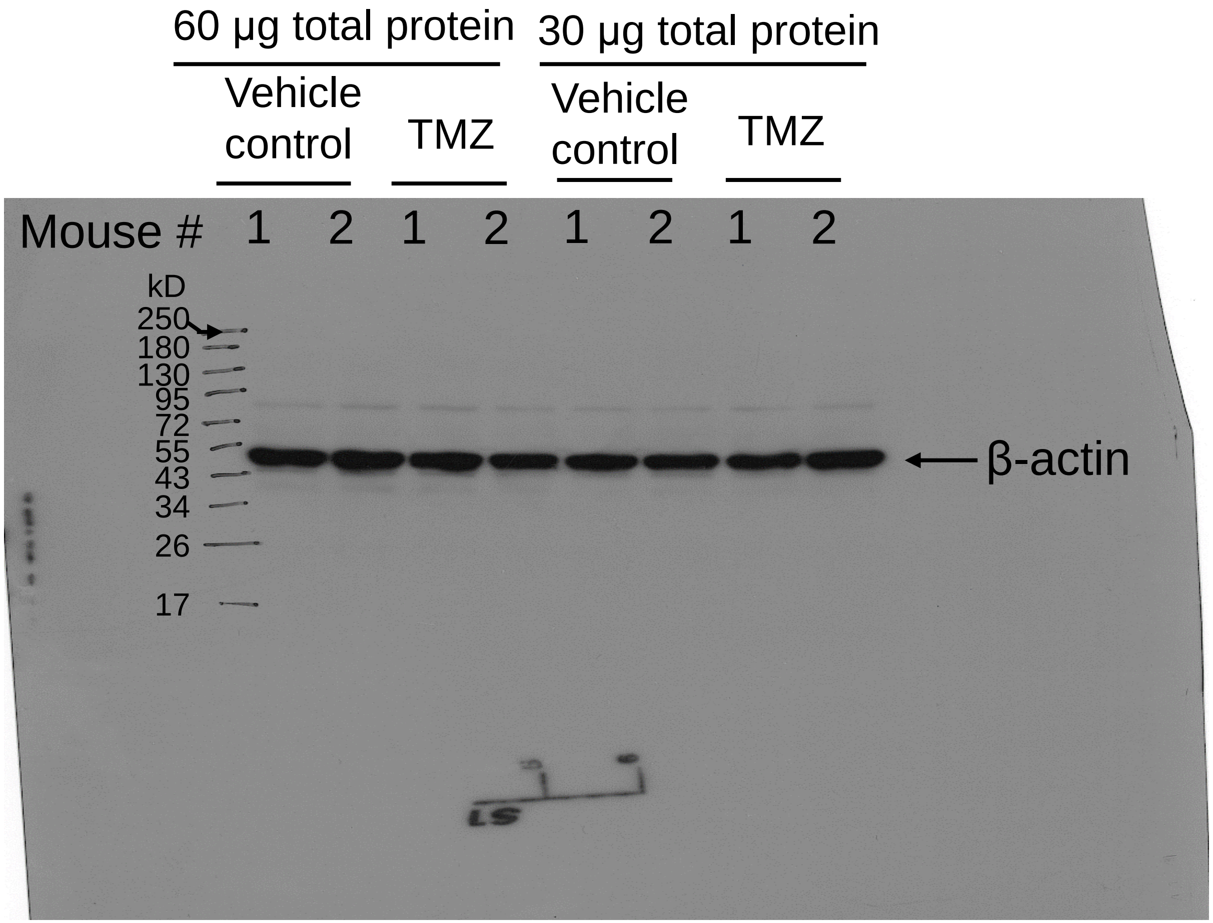
60 µg total protein
30 µg total protein
Vehicle control
TMZ
Vehicle control
TMZ
Mouse #
1
2
1
2
1
2
1
2
kD
250
180
130
95
72
55
43
34
26
17
β-actin

C

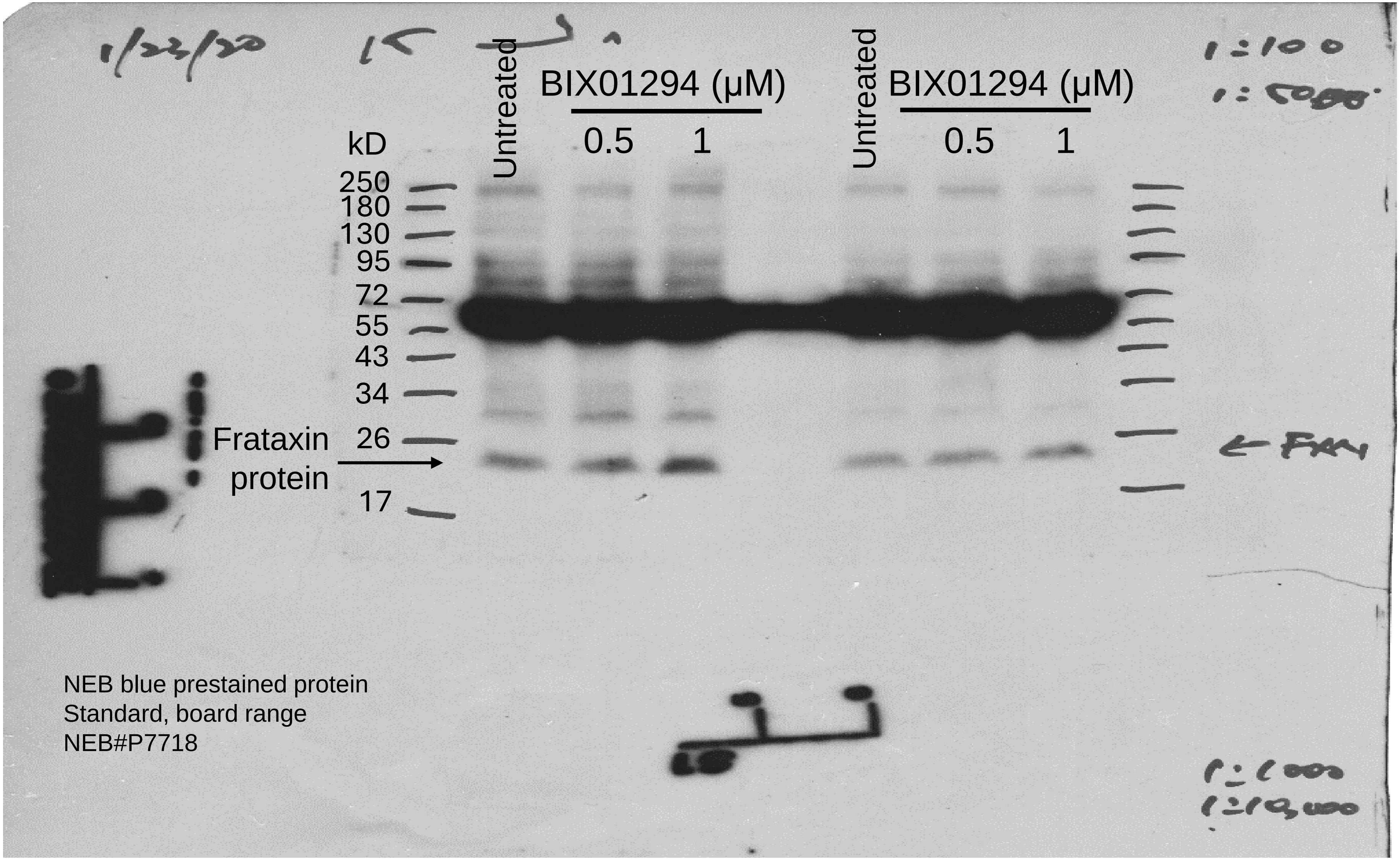
Untreated
BIX01294 (μM)
0.5
1
Untreated
BIX01294 (μM)
0.5
1
kD
250
180
130
95
72
55
43
34
26
17
Frataxin protein
1:100
1:5000
1:1000
1:10,000
← FXN
NEB blue prestained protein Standard, board range NEB#P7718

D

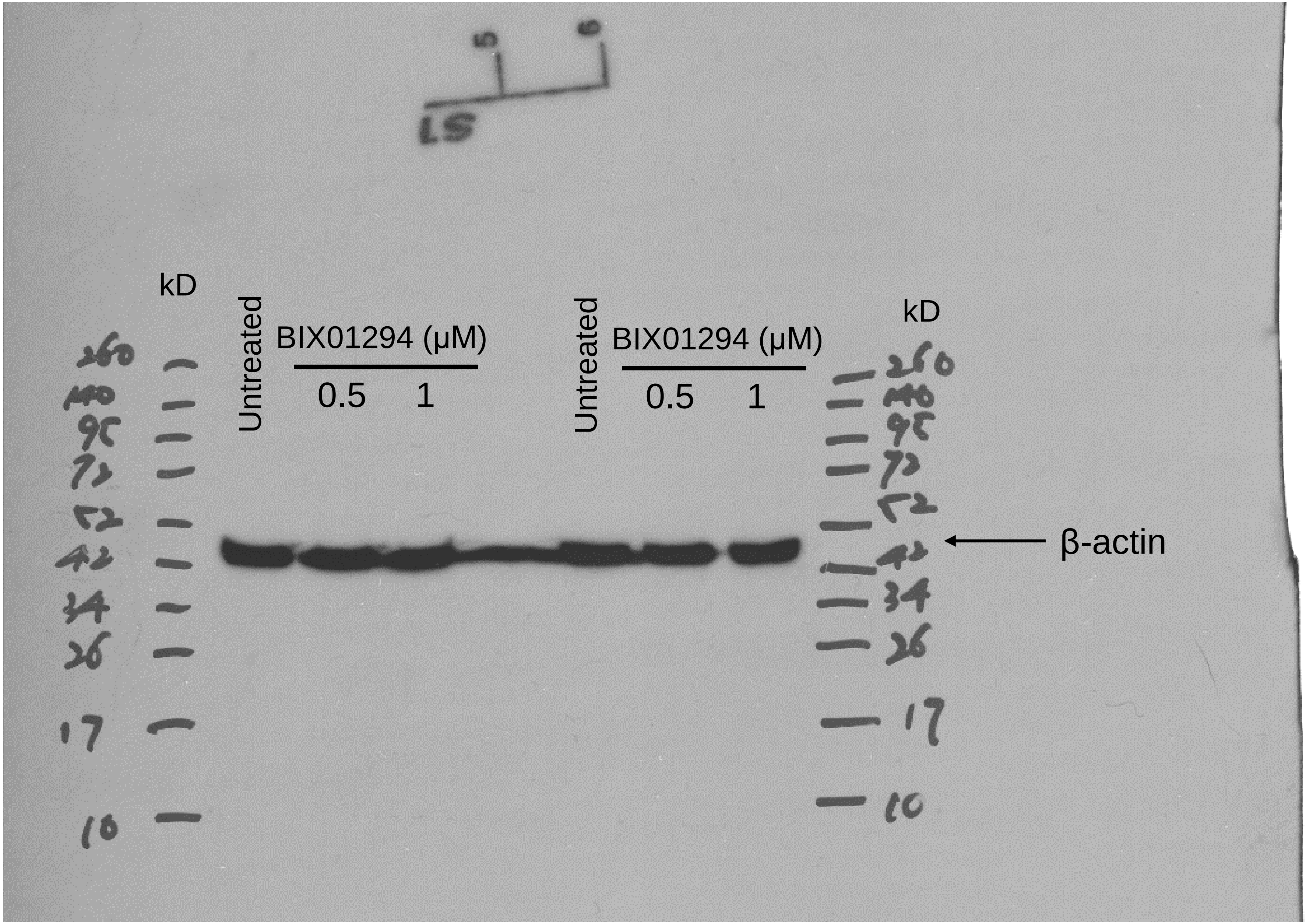
kD
Untreated
BIX01294 (μM)
0.5
1
Untreated
BIX01294 (μM)
0.5
1
kD
β-actin
260
140
95
72
52
42
34
26
17
10

Supplementary Figure Legends

Supplementary Figure S1. Schematic diagram for experiments using FRDA transgenic mice. 12-week-old FRDA transgenic mice were treated with TMZ (25 mg/kg body weight) via oral gavage (three times per week) for three months. Mouse brain tissue was collected 1 day after the last treatment for subsequent analysis, as illustrated.

Supplementary Figure S2. Identification of primary cultured cerebellar neuronal cells from FRDA transgenic mice and differentiated human FRDA neural cells. (A) Primary cultured cerebellar neuronal cells were identified by staining the mouse cerebellar granule and Purkinje neuronal cell markers, Zic2, CALB1, and the general neuronal marker, Tuj1, with IF. (B) Differentiated human FRDA neuronal cells were identified by staining human neuronal markers Tuj1 and MAP2 in cells with IF. For all the experiments, DAPI (0.1 μg/ml) was used to stain the nucleus, and cell images were captured at a scale of 100 μm.

Supplementary Figure S3. Strand break-mediated DNA modification (SBDM) profiling assay for detecting single-strand breaks on GAA repeats at the *FXN* gene. Alkylated DNA bases induced by TMZ and oxidized DNA bases that are located at various units of the GAA repeats at the *FXN* gene are removed by endogenous alkyladenine DNA glycosylase (AAG) and 8-oxoguanine DNA glycosylase (OGG1) in FRDA neural cells, leaving abasic sites, which are then converted to ssDNA breaks by AP endonuclease 1 (APE1). Subsequently, primer 1 is annealed to the strands with the breaks and extended by a DNA polymerase, generating different sizes of double-strand DNA fragments with blunt ends that are ligated with barcode DNA. The DNA fragments are amplified and labeled by a 6-fluorescein (6-FAM) fluorophore through PCR with the barcode primer and 6-FAM-tagged-primer 2. Amplified DNA fragments are separated by capillary electrophoresis, and their sizes are determined by DNA fragment analysis with the GeneMapper V.4 software. The locations of base lesions in GAA repeats are determined by the sizes of the fragments after subtracting the sizes of primer 1, barcode DNA, and 3'-flanking regions of the repeats, and the unique profiles of base lesions are determined.

Supplementary Figure S4. The original Western blot gel data of frataxin and β-actin protein level in FRDA mouse brain tissue and differentiated human FRDA neural cells. (A) The original gel data of frataxin protein level in FRDA mouse brain tissue without and with the treatment of TMZ for Figure 1B. (B) The original gel data of β-actin protein (loading control) level in FRDA mouse brain tissue without and with the treatment of TMZ for Figure 1B. (C) The original gel data of frataxin protein level in differentiated human FRDA neural cells treated with BIX-01249 for Figure 4B. (D) The original gel data of β-actin protein (loading control) level in differentiated human FRDA neural cells treated with BIX-01249 for Figure 4B.
